# Modeling of chemical inhibition from amyloid protein aggregation kinetics

**DOI:** 10.1186/2050-6511-15-9

**Published:** 2014-02-27

**Authors:** José Antonio Vázquez

**Affiliations:** 1 Grupo de Reciclado e Valorización de Residuos (REVAL), Instituto de Investigacións Mariñas (IIM-CSIC), C/ Eduardo Cabello 6, CP36208 Vigo, Spain

**Keywords:** Protein fibrillation kinetics, Mathematical modeling, Sigmoid bivariate equation

## Abstract

**Backgrounds:**

The process of amyloid proteins aggregation causes several human neuropathologies. In some cases, *e.g*. fibrillar deposits of insulin, the problems are generated in the processes of production and purification of protein and in the pump devices or injectable preparations for diabetics. Experimental kinetics and adequate modelling of chemical inhibition from amyloid aggregation are of practical importance in order to study the viable processing, formulation and storage as well as to predict and optimize the best conditions to reduce the effect of protein nucleation.

**Results:**

In this manuscript, experimental data of insulin, Aβ42 amyloid protein and apomyoglobin fibrillation from recent bibliography were selected to evaluate the capability of a bivariate sigmoid equation to model them. The mathematical functions (logistic combined with Weibull equation) were used in reparameterized form and the effect of inhibitor concentrations on kinetic parameters from logistic equation were perfectly defined and explained. The surfaces of data were accurately described by proposed model and the presented analysis characterized the inhibitory influence on the protein aggregation by several chemicals. Discrimination between true and apparent inhibitors was also confirmed by the bivariate equation. EGCG for insulin (working at pH = 7.4/T = 37°C) and taiwaniaflavone for Aβ42 were the compounds studied that shown the greatest inhibition capacity.

**Conclusions:**

An accurate, simple and effective model to investigate the inhibition of chemicals on amyloid protein aggregation has been developed. The equation could be useful for the clear quantification of inhibitor potential of chemicals and rigorous comparison among them.

## Background

The aggregation and fibrillation of proteins has been commonly associated with numerous degenerative disorders in humans including Alzheimer’s, Parkinson’s, prion’s, diabetes type II and Huntington’s diseases [1-5]. These proteins, called amylodogenics, are also involved in production, purification and formulation interferences of biotechnology and pharmacologic processes [6,7]. The phenomenon of protein aggregation, led by nucleation-dependent polymerization mechanism, consists in the formation of amyloid fibrils formulated with β-sheet structures in cross-β-sheet arrangement producing insoluble aggregates [8-10]. For example, insulin peptide also generates fibrillar structures under specific conditions such as low pH, high temperatures or organic solvents dilution [11-13]. Those phase transformations are not observed *in vivo* but are very common, for example, in the clinical preparations of insulin for diabetics [14]. Human amyloid proteins (Aβ) are peptides of rather 39-42 residues. Aβ40 contains 40 amino acids and Aβ42 is the major isoform in the Aβ peptides with 42 residues polypeptide chain and it is the responsible of amyloid plaques generated in Alzheimer’s disorder [15,16].

In general, much of the success in the future application of chemicals to inhibit the *in vivo* formation of such fibrils is dependent on the correct modelling and evaluation of *in vitro* kinetic data in order to establish protocols of action, effectiveness of molecules and dose-optimization of compounds. The kinetic description of amyloid protein aggregation based on mechanistic and thermodynamic approaches have been extensively studied [17-22] and an ample number of plausible mechanisms of nucleation and fibrillation have been proposed [23,24]. So remarkable, two new and similar proposal: “Ockham's razor”/minimalistic and Crystallization-like Model, have been recently developed. Both have solid biophysical basis and they were successfully applied to describe and explain the experimental data of different amyloid protein aggregation [25-28]. However, the combined effect of anti-aggregation protein agents, *e.g.* surfactants, osmolytes and food additives, on time-dependent responses has not been modelled by that way or by means of empirical equations.

Although the use of empirical sigmoid equations, mainly the logistic equation, does not provide a direct explanation of the molecular steps that underlie in the generation of fibrils, it is a robust tool to examine protein aggregation kinetic data and to address all the phases of the process [4,29,30]. In addition, other authors assimilated the parameters from logistic [20] to those obtained by an autocatalytic mechanism [23,27]. Nonetheless, that equation is always formulated without the parameters (fibrillation rate and lag phase) in an explicit form hindering the estimation of their statistical error.

In the present work, the capability of fit and experimental data predictability of a sigmoid bivariate model that simulates the growth of aggregation process on different proteins along with the effects of inhibitory chemicals on the kinetic parameters is explored in selected cases obtained from the literature. The results reveal its efficacy and validity to analyze the most relevant parameters that describe geometrically and macroscopically the mentioned process.

## Methods

### Experimental data

Amyloid protein aggregation data were collected from results previously reported in the bibliography and digitized from the published curves using GetData Graph Digitizer 2.24. The kinetics of insulin inhibition induced by (−)-epigallocatechin-3-gallate (EGCG) were selected from Wang et al. [31], methylglyoxal effects were collected from Oliveira et al. [32] and those produced by 1,2-diheptanoyl-sn-glycero-3-phosphocholine (di-C7-PC) were described in Wang et al. [33]. Two conditions of EGCG affecting to fibrillation kinetics were used: EGCG_1 (case 1) studied at pH = 2.0/T = 60°C and EGCG_2 (case 2) at pH = 7.4/T = 37°C.

On the other hand, the aggregation kinetics of Aβ42 amyloid protein inhibited by apigenin and taiwaniaflavone were selected from Thapa et al. [16] and the data affected by ectoine and hydroxyectoine from Kanapathipillai et al. [15]. Finally, apomyoglobin fibrillation experiments were published in Vilasi et al. [34]. The datasets were obtained by the two most common methods used to probe amyloid formation in vitro, the increment in light scattering of the protein solution due to insolubilization, and the increase in ThT fluorescence due to amyloid binding.

### Mathematical modelling

The model developed to simulate the process of aggregation and hence insulin fibrillation was defined by a bivariate equation. Such model is based on the combination of Weibull function as chemical-concentration model [35,36] modifying the most important parameters of the reparameterized logistic equation [37] used for aggregation description. This expression (1) has been successfully used, in recent works, to evaluate the inhibitory effect of organic acids and heavy metals on the growth of various bacteria [38,39]. Its mathematical form is as follows (see also Appendix section):

(1) $$X=\frac{X_{m•}}{1+\exp\;\left[2+\frac{4v_{m•}}{X_{m•}}\left(\lambda_{•}-t\right)\right]}\;;\;\mathrm{where}:$$

$$X_{m•}=X_{m}\left\{1-K_{x}\left[1-\exp\;\left(-\ln2\;{\left(\frac{C}{m_{x}}\right)}^{a_{x}}\right)\right]\right\}$$

$$v_{m•}=v_{m}\left\{1-K_{v}\left[1-\exp\;\left(-\ln2\;{\left(\frac{C}{m_{v}}\right)}^{a_{v}}\right)\right]\right\}$$

$$\lambda_{•}=\lambda \left\{1+K_{\lambda }\left[1-\exp\;\left(-\ln2\;{\left(\frac{C}{m_{\lambda }}\right)}^{a_{\lambda }}\right)\right]\right\}$$

where, *v*_*m*_ is the maximum aggregation rate, *X*_*m*_ is the maximum aggregation growth, *λ* is the lag phase and *C* is the chemical concentration. The meanings of other symbolic notations as well as the corresponding units are summarized in Table 1. In the experimental data chosen, the dependent variable of response or time-dependent signals (*X*) to detect amyloid protein aggregation were absorbance at 600 nm (data obtained from Wang et al. [31]), relative ThT fluorescence intensity (%) (data from Wang et al. [33]) and ThT fluorescence intensity at 482 nm or 490 nm (data obtained from Thapa et al. [16]; Kanapathipillaia et al. [15]; Oliveira et al. [32] and Vilasi et al. [34]).

**Table 1:** Symbolic notations used and corresponding units

Insulin aggregation kinetics measured by absorbance or fluorescence	
*X* :	Amyloid aggregation growth measured as absorbance at 600 nm, relative ThT fluorescence intensity (%) and ThT fluorescence intensity at 482 nm or 490 nm. Units: absorbance units (AU) or (%).
*t* :	Time. Units: h or d
*X*_*m*_ :	Maximum aggregation growth. Units: AU or %
*v*_*m*_ :	Maximum aggregation rate. Units: AU h^−1^, AU d^−1^ or % h^−1^
*λ* :	Lag phase. Units: h or d
*X*_*m*_*•* :	Maximum insulin aggregation affected by chemical agent. Units: AU or %
*v*_*m*_*•* :	Maximum insulin aggregation rate affected by chemical agent. Units: AU h^−1^, AU d^−1^ or % h^−1^
*λ*• :	Lag phase affected by chemical agent. Units: h or d
Concentration effects on insulin aggregation kinetics	
*C* :	Concentration of chemical agent. Units: mM or μM
*K*_*x*_ :	Maximum response affecting on *X*_*m*_. Dimensionless
*m*_*x*_ :	Concentration corresponding to the semi-maximum response affecting on *X*_*m*_. Units: mM or μM
*a*_*x*_ :	Shape parameter affecting on *X*_*m*_. Dimensionless
*K*_*v*_ :	Maximum response affecting on *v*_*m*_. Dimensionless
*m*_*v*_ :	Concentration corresponding to the semi-maximum response affecting on *v*_*m*_. Units: mM or μM
*a*_*v*_ :	Shape parameter affecting on *v*_*m*_. Dimensionless
*K*_*λ*_ :	Maximum response affecting on *λ*. Dimensionless
*m*_*λ*_ :	Concentration corresponding to the semi-maximum response affecting on *λ*. Units: mM or μM
*a*_*λ*_ :	Shape parameter affecting on *λ*. Dimensionless

Additionally, a global parameter (*EC*_*50,τ*_) was also selected for the overall description of chemical effects on fibrillation growth studies according the algebraic steps previously described [38]. This parameter was defined as the chemical concentration (in mM) that reduces the aggregation by 50% compared to that produced by the control without agent at time (τ) which also reduces the maximum aggregation by 50%.

### Numerical methods and statistical analysis

The fitting procedures and parametric estimates from the experimental results were performed by minimizing the sum of quadratic differences between the observed and model-predicted values using the nonlinear least-squares (quasi-Newton) method provided by the ‘*Solver*’ macro from Microsoft Excel spreadsheet. The confidence intervals of the best-fit values for the parametric estimates (Student’s t test, α = 0.05), consistency of the mathematical models (Fisher’s F test; *p* < 0.05) and covariance and correlation matrices were calculated using the ‘*SolverAid*’ macro, which is freely available from Levie’s Excellaneous website http://www.bowdoin.edu/~rdelevie/exellaneous/. An example of the type of Excel spreadsheet used for modeling (Ab42amyloid apigenin case) was provided as Additional file 1. These statistical procedures and residual analysis (Durbin-Watson test) were confirmed and evaluated, respectively, by DataFit 9 (Oakdale Engineering, Oakdale, PA). Moreover, bias and accuracy factors of fittings (*B*_*f*_ and *A*_*f*_, respectively) were calculated according to the expressions reported by Ross [40].

## Results and discussion

### Characteristics and simulations of bivariate model

**Figure 1:**
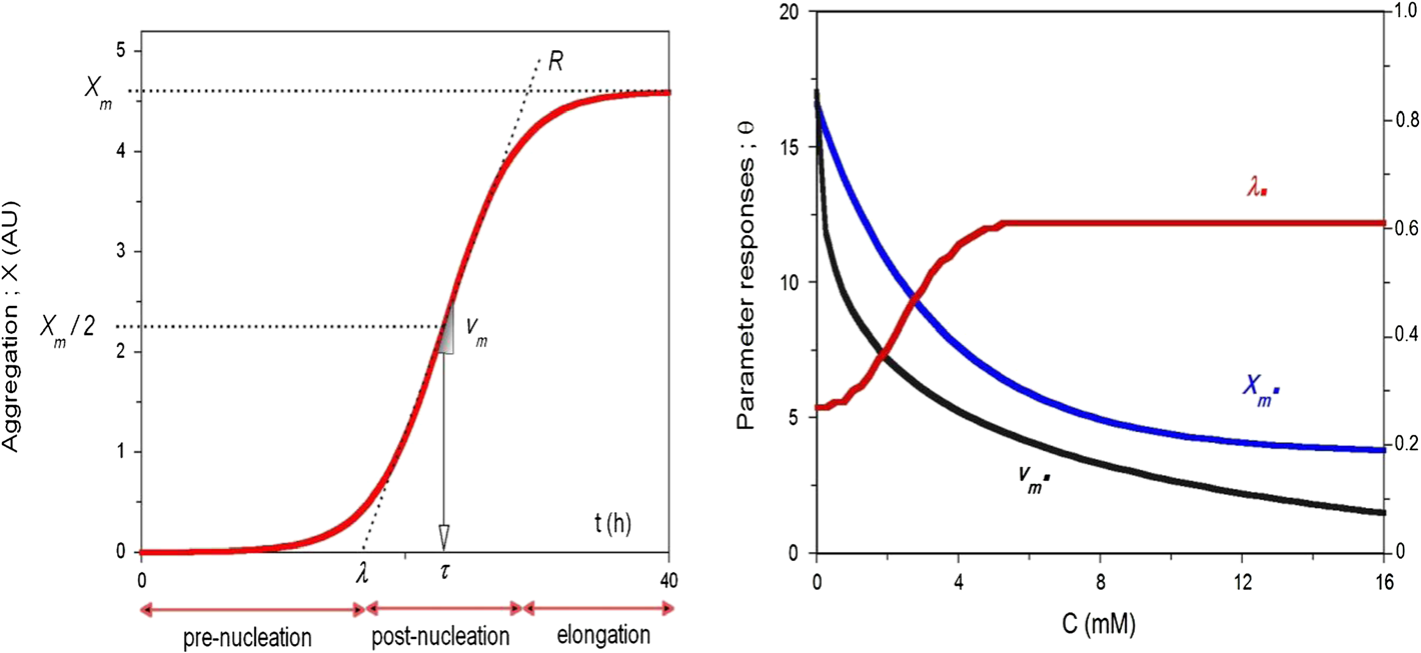
**Left, Graphical description of the kinetic parameters (*X***_***m***_**, *λ*, *v***_***m***_**and τ) from the logistic equation (**A.8**) and the corresponding aggregation phases: pre-nucleation, post-nucleation and elongation.** Right, Simulations of the most common profiles for the parameters (*X*_*m*_•, *λ*•, *v*_*m*_•), affected by chemical concentration, using the Weibull equations (A.15).

In the description of amyloidogenic fibrillation growths, the logistic equation used to formalize their kinetic profiles is always formulated through an explicit expression based on the time required to reach 50% of the maximal aggregation (*x*_*0*_) and a time parameter (τ), although the initial lag phase and the aggregation rate are commonly the most important parameters that need to be calculated [30]. In this work, the logistic equation was reparameterized to make the lag phase (*λ*) and the maximum aggregation rate (*v*_*m*_), which represents the overall aggregation process rate, explicit (Figure 1). This last coefficient more adequately characterizes the reaction rate of fibrillation than the parameter conventionally used (*k*_*app*_, defined as specific or apparent rate) because it is less sensitive to experimental error and because it is a more efficient to describe the global rate of the kinetic process [41]. Additionally, the *v*_*m*_ calculated from the experimental data obtained by Sabaté et al. [42] for yeast prion proteins follows the Arrhenius dependence with temperature (data not shown).

In contrast, the fit using the reparameterized functions can be used to easily calculate the confidence intervals of the parameters. The algebraic steps required to obtain the corresponding reparameterization of *λ* and *v*_*m*_ from the logistic equation are detailed in the Appendix. Thus, the parameters from this equation define all of the aggregation kinetic phases [19]: pre-nucleation, which is characterized by *λ*, post-nucleation, which is represented by *v*_*m*_ or τ, and elongation, which is determined by *X*_*m*_.

The absolute correlation between the lag phase and the aggregation growth was recently demonstrated in several sets of protein data [43]. Indeed, this relationship is obvious because the specific or apparent rate (*k*_*app*_ or *k*_*g*_, depending on the authors) is inversely proportional to the lag phase based the following expression when the logistic model (1) is applied [44]:

$$\lambda =\tau -\frac{2}{k_{\mathrm{app}}}$$

**Figure 2:**
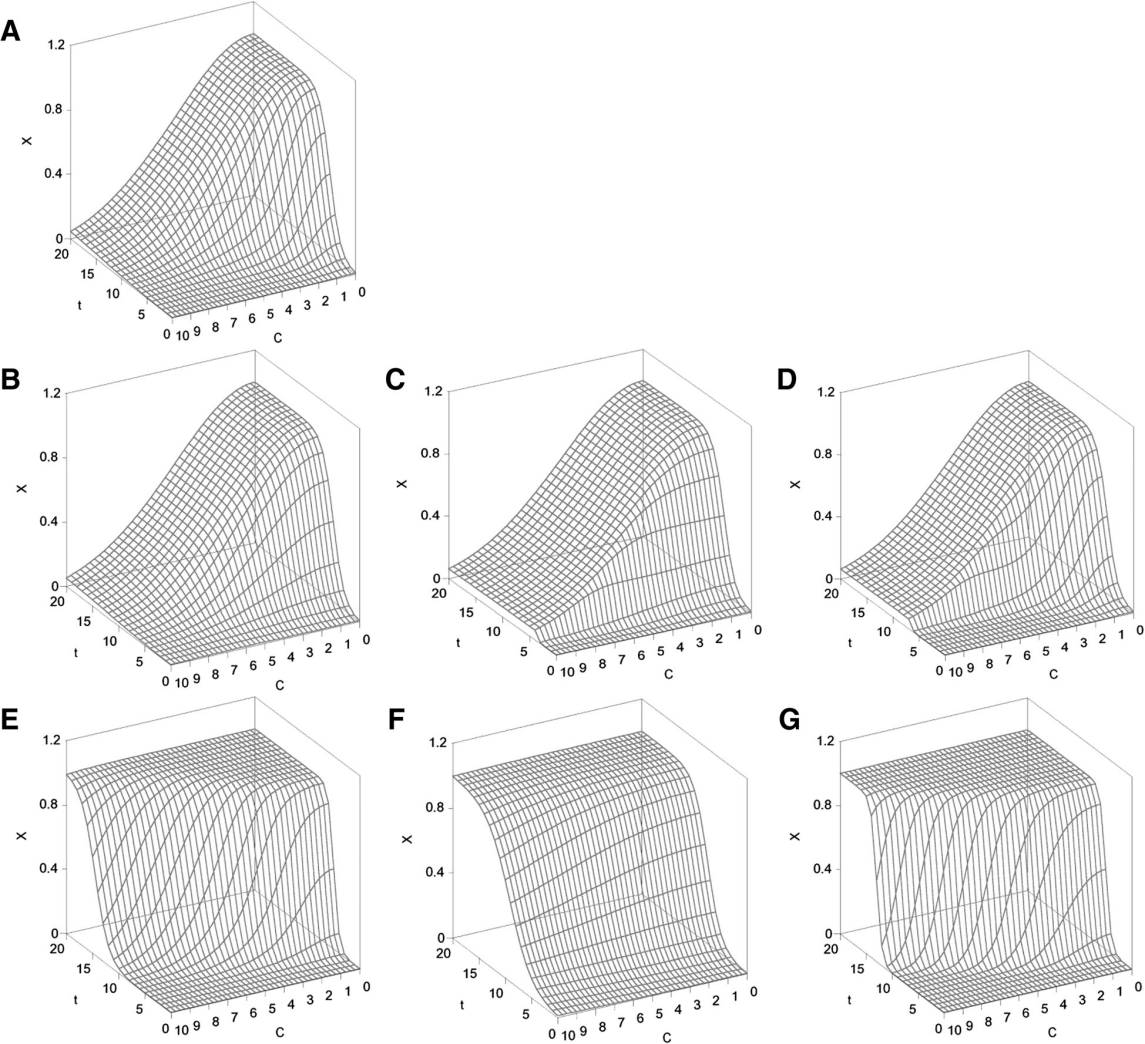
**Profiles obtained by simulation under the numerical conditions specified in Table**2**using the equation (1) and that representing all the theoretical kinetics of protein aggregation process. A**: all the parameters from logistic equation (*X*_*m*_, *v*_*m*_ and *λ*) are affected by chemical concentration; **B**: parameters (*X*_*m*_ and *v*_*m*_) are modified by chemical; **C**: only the parameter *X*_*m*_ is affected by chemical; **D**: parameters (*X*_*m*_ and *λ*) are modified by chemical; **E**: parameters (*v*_*m*_ and *λ*) are affected by chemical; **F**: only the parameter *v*_*m*_ is modified by chemical; **G**: only the parameter *λ* is affected by chemical. In all cases, time (*t*), aggregation response (*X*) and chemical concentration (*C*) are simulated with arbitrary units.

Figure 2 shows an illustrative set of simulations that were developed under the numerical conditions described in Table 2. In these simulations, all of the possible sigmoid effects of the chemical concentration on the logistic parameters are defined. Weibull’s equation, which was adequately configured to simulate the dose-response trends, is a mathematical tool that provides excellent predictions for varied experimental profiles from different scientific fields [45,46]. In the present context, the flexibility of this equation to describe different chemical-concentration relationships as linear, sigmoid, exponential, or hyperbolic curves depending on the numerical values of the parameters is particularly valuable. The combination of both of these mathematical resources (1) generates a wide variety of theoretical casuistries that will be verified using the experimental data selected below.

**Table 2:** **Arbitrary numerical values defined for the simulations of Figure**1**(A, B, C, D, E, F and G) according to the parameters defined in the equation (**1**)**

		**Simulation conditions**						
**Parameters**		**A**	**B**	**C**	**D**	**E**	**F**	**G**
Aggregation growth	*X*_*m*_	1.00	1.00	1.00	1.00	1.00	1.00	1.00
	*v*_*m*_	0.25	0.25	0.25	0.25	0.50	0.20	0.50
	*λ*	3.00	3.00	3.00	3.00	4.00	5.00	4.00
Effect on *X*_*m*_	*K*_*x*_	1.00	1.00	1.00	1.00	-	-	-
	*m*_*x*_	5.00	5.00	5.00	5.00	-	-	-
	*a*_*x*_	2.00	2.00	2.00	2.00	-	-	-
Effect on *v*_*m*_	*K*_*v*_	1.00	1.00	-	-	0.60	0.60	-
	*m*_*v*_	4.00	4.00	-	-	3.00	8.00	-
	*a*_*v*_	2.00	2.00	-	-	2.00	2.00	-
Effect on *λ*	*K*_*λ*_	1.00	-	-	1.00	4.00	-	4.00
	*m*_*λ*_	2.00	-	-	2.00	10.00	-	10.00
	*a*_*λ*_	2.00	-	-	2.00	2.00	-	2.00

### Inhibitory effect of EGCG on insulin aggregation

**Figure 3:**
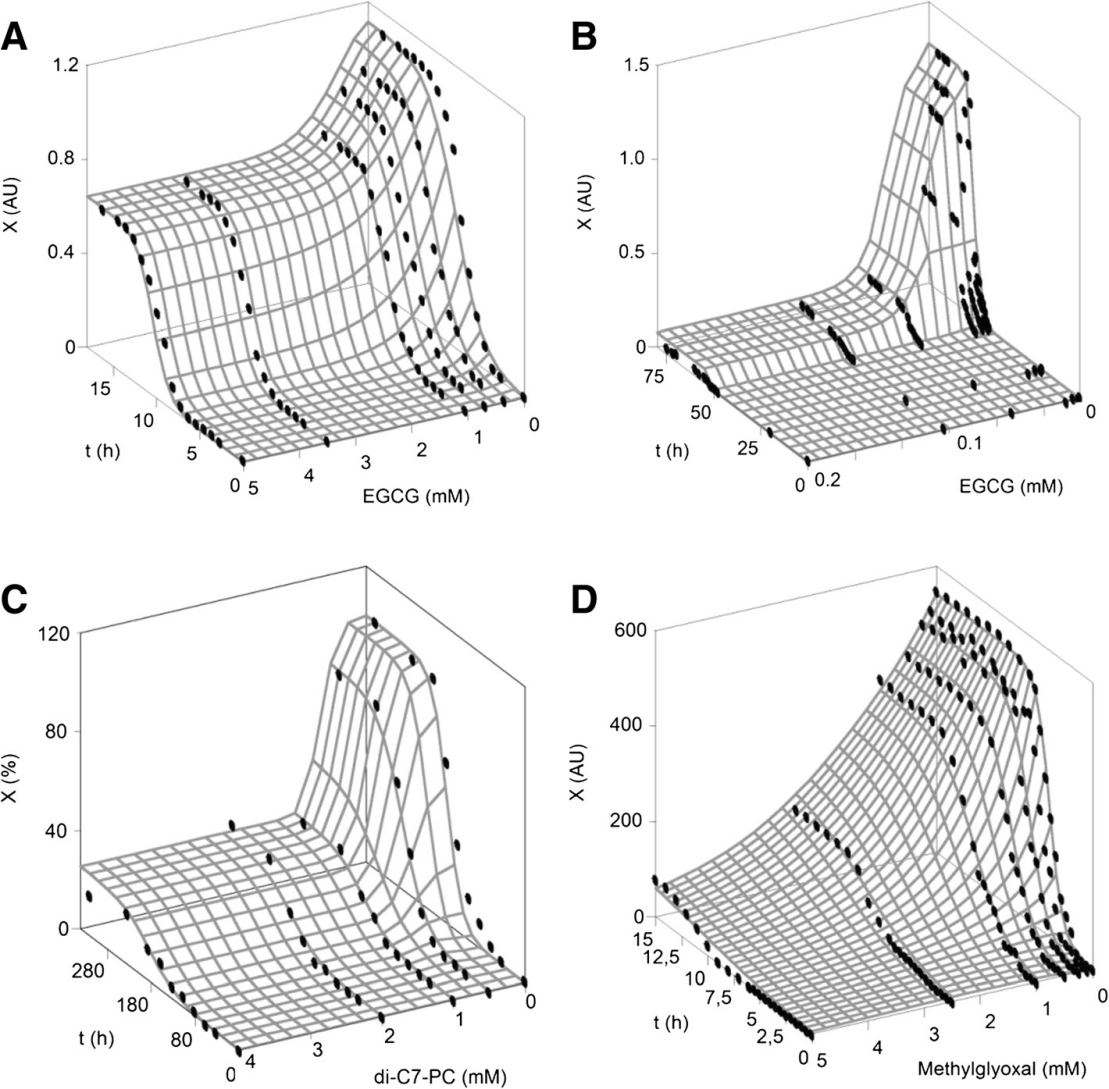
**Insulin fibrillation kinetics at different concentrations of EGCG, di-C7-PC and methylglyoxal (points) and fittings to equation (1) (surface).***X*: aggregation data measured by absorbance at 600 nm (AU), ThT fluorescence intensity at 482 nm (AU) or ThT fluorescence intensity (%). **A**: experimental data of EGCG obtained at pH = 2.0 and T = 60°C. **B**: experimental data of EGCG obtained at pH = 7.4 and T = 37°C. **C**: di-C7-PC data. **D**: methylglyoxal data.

The representation of the experimental data from selected cases and surfaces predicted by equation (1) are depicted in Figure 3A and B. The results of the parameter estimations and the statistical analysis of the fit for the EGCG influence on insulin fibrillation are summarized in Table 3. Both datasets were accurately fitted with equation (1) to obtain *R*^*2*^_*adj*_ values greater than 0.98. Moreover, the *p*-values obtained from Fisher’s F test indicated the complete consistency and robustness of the equation to the adjustment of the experimental patterns. The residual analysis through the Durbin-Watson test revealed a lack of residual autocorrelation and therefore a random distribution (data not shown).

**Table 3:** **Parametric estimates and confidence intervals (α = 0.05) from the equation (**1**) applied to the aggregation insulin increased data influenced by EGCG_1, EGCG_2, di-C7-PC and methylglyoxal concentrations**

**Parameters**		**EGCG_1**	**EGCG_2**	**di-C7-PC**	**Methylglyoxal**
Aggregation model	*X*_*m*_ (AU or %)	1.12±0.03	1.28±0.06	99.85±4.91	541.20±11.38
	*v*_*m*_ (AU h^−1^ or % h^−1^)	0.18±0.01	0.25±0.04	1.55±0.27	219.68±20.98
	*λ* (h)	4.78±0.28	53.34±0.28	124.62±5.86	2.92±0.12
Effect on *X*_*m*_	*K*_*x*_	0.43±0.03	0.94±0.02	0.75±0.05	1.00±0.44
	*m*_*x*_ (mM)	0.68±0.08	0.01±0.00	0.65±0.12	1.80±1.25
	*a*_*x*_	1.65±0.45	0.96±0.20	4.04±2.37	0.99±0.23
Effect on *v*_*m*_	*K*_*v*_	NS	NS	0.86±0.04	0.99±0.01
	*m*_*v*_ (mM)	NS	NS	0.47±0.06	0.42±0.07
	*a*_*v*_	NS	NS	2.51±2.35	0.74±0.08
Effect on *λ*	*K*_*λ*_	0.81±0.10	NS	NS	NS
	*m*_*λ*_ (mM)	0.50±0.08	NS	NS	NS
	*a*_*λ*_	0.98±0.24	NS	NS	NS
	*EC*_*50,τ*_ (mM)	0.44	0.006	0.47	0.33
	*τ* (h)	7.65	55.02	150.68	3.92
	*p*-value	<0.001	<0.001	<0.001	<0.001
	*B*_*f*_	0.88	0.80	1.00	1.04
	*A*_*f*_	1.23	1.40	1.18	1.15
	*R*^*2*^_*adj*_	0.992	0.977	0.984	0.974

Statistical values of adjusted coefficient of multiple determination (*R*^*2*^_*adj*_) and *p*-values from Fisher’s F-test (α = 0.05). *B*_*f*_ and *A*_*f*_ are the bias and accuracy factor, respectively. NS: non-significant.

At an acidic pH and a high temperature, the effect of EGCG on the kinetic parameters was not significant for the maximum aggregation rate but statistically significant for *X*_*m*_ and the lag phase. As can be observed in Figure 3A, the slopes of the sigmoid curves were parallel to the influence of the EGCG concentration, and the surface can be assimilated to that simulated in Figure 2D but with less effect on *X*_*m*_. These results show a longer pre-nucleation phase, which indicates that EGCG blocks the formation of the seeding nuclei without changing the fibril elongation rate (post-nucleation). This behaviour is consistent with the definition reported by Martins [26] who denominates the compounds that modify kinetic parameters (for instance in the present case, *v*_*m*_ and/or *λ*) as true inhibitors.

In contrast, different surface and parametric responses were observed for EGCG_2 up to a concentration of 0.2 mM polyphenol with only significant modifications in the maximum aggregation growth parameter (Figure 3B). Under experimental conditions of higher pH and lower T, EGCG acts as an apparent inhibitor that only alters the thermodynamic properties of aggregation process [26]. Higher concentrations of EGCG (from 0.35 to 0.7 mM) led to an increase in the fibrillation response more than the inhibition of the amyloid process [31]. These experimental points were deleted in this mathematical analysis because biphasic aggregation data with profiles of decreasing and increasing values of *X*_*m*_ cannot be fitted by the proposed equation or by any other simple equation.

The global description of chemical inhibition was calculated by means of a single index: *EC*_*50,*τ_ (Table 3). This parameter can be defined as a summary of all of the effects on the aggregation kinetics observed at the time required to reach the semi-maximum aggregation growth. Thus, the obtained value of this parameter can be used for the comparison and the evaluation of the application of chemicals to reduce the protein fibrillation. The results demonstrated that the most inhibitory conditions for EGCG are established at neutral pH and a lower temperature (case 2), but these conditions require a longer time (*τ*) to achieve the parameter *EC*_*50,*τ_.

### Inhibitory effect of di-C7-PC and methylglyoxal on insulin aggregation

Figure 3C displays the surface described by equation (1) and data on the insulin kinetics in the presence of a surfactant. The parameter values and the statistical characteristics of the modeling were also determined (Table 3). All of the statistical results revealed good agreement and accuracy between the observed and the predicted values, the consistency of the equation, and a lack of bias during the fitting process.

Regarding the effects, the two kinetic parameters *v*_*m*_ and *X*_*m*_ were significantly affected by di-C7-PC, whereas the lag phase was not modified by the surfactant (Student’s t test, α = 0.05), as determined in the simulated conditions defined by Figure 2B. This result is in agreement with data reported for different systems of effector/protein, such as copper/β-amyloid [47] or glutathione/hen egg-white lysozyme, although these data were obtained using a non-mathematical approach [48]. The value of *EC*_*50,*τ_ was similar to that obtained for EGCG (case 1) for a lower initial concentration of insulin, but a much longer time was required to obtain this parametric value (150.68 h).

Similar inhibitory responses were also observed with methylglyoxal; thus, *v*_*m*_ and *X*_*m*_ were the kinetic parameters inhibited by this aldehyde form of pyruvic acid (Table 3 and Figure 3D). Under an initial insulin concentration (3 g/L) that was higher than the initial EGCG concentration, methylglyoxal effectively reduced the aggregation growth (0.33 mM) within a shorter time (3.9 h). Both chemicals (di-C7-PC and methylglyoxal), under the experimental conditions assayed, showed similar features of true inhibitors [26]. The application of the proposed methodology may help the design of optimal strategies for the reduction of the fibril deposition of insulin in commercial preparations.

### Inhibitory effect of mono and biflavonoids on Aβ42 amyloid protein

**Figure 4:**
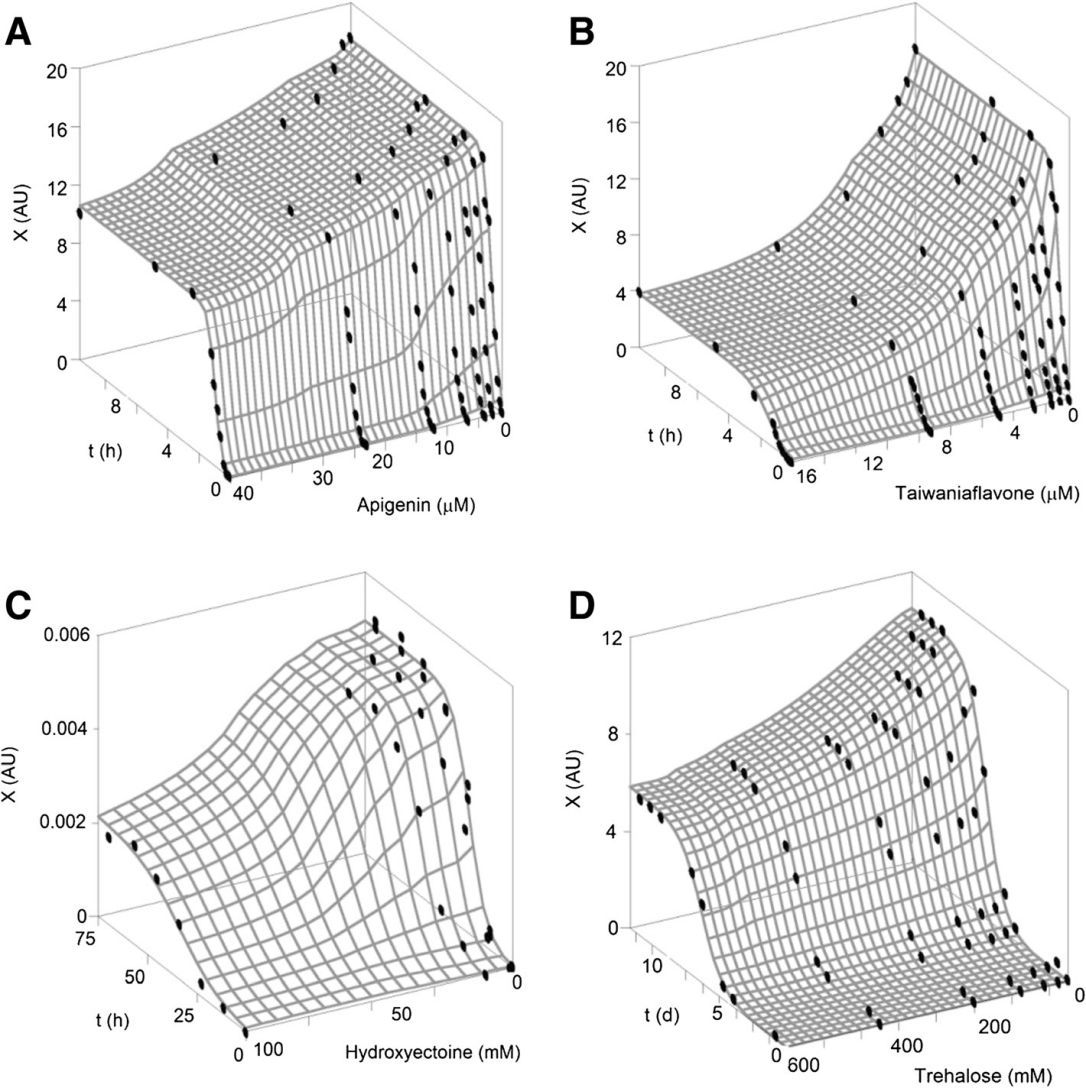
**Amyloid protein aggregation kinetics at different concentrations of apigenin, ectoine, taiwaniaflavone and trehalose (points) and fittings to equation (1) (surface).***X*: aggregation data measured by ThT fluorescence intensity at 482 nm or 490 nm (AU). **A**: experimental data of apigenin. **B**: taiwaniaflavone data. **C**: experimental data of hydroxyectoine. **D**: data of trehalose affecting apomyoglobin kinetics.

The dependence of the kinetic parameters of fibrillation on the apigenin and taiwaniaflavone concentrations is represented in Figures 4A and B. Both response surfaces were similar to that defined by the simulation shown in Figure 2A. The three parameters (*v*_*m*_, *X*_*m*_, and *λ*) were clearly and significantly modified by these chemical doses (Table 4), which show a longer pre-nucleation phase, a lower elongation rate (post-nucleation), and a lower elongation stage. Such responses confirmed their chemical features of true inhibitors, being the only case in which both kinetic parameters (*v*_*m*_ and *λ*) significantly affected aggregation process [26]. All of the statistical tests corroborated the perfect agreement between the experimental and the theoretical data (*e.g., R*^*2*^_*adj*_ > 0.99). The bias and accuracy factors (*B*_*f*_ and *A*_*f*_) were also indicative of the good fitting quality obtained with model (1).

**Table 4:** **Parametric estimates and confidence intervals (α = 0.05) from the equation (**1**) applied to the aggregation of Aβ42-amyloid protein increased data influenced by apigenin, ectoine, hidroxyectoine and taiwaniaflavone concentrations**

**Parameters**		**Apigenin**	**Ectoine**	**Hidroxyectoine**	**Taiwaniaflavone**	**Trehalose**
Aggregation model	*X*_*m*_ (AU)	17.43±0.50	(5.07±0.23) × 10^−3^	(4.96±0.24) × 10^−3^	16.55±0.55	10.49±0.28
	*v*_*m*_ (AU h^−1^ or AU d^−1^)	16.10±1.49	(0.24±0.05) × 10^−3^	(0.30±0.08) × 10^−3^	17.01±2.09	3.31±0.33
	*λ* (h or d)	0.25±0.03	11.60±2.26	13.50±2.31	0.27±0.06	5.76±0.14
Effect on *X*_*m*_	*K*_*x*_	0.41±0.06	1.00±0.99	1.00±0.90	0.78±0.05	0.44±0.03
	*m*_*x*_ (mM or μM)	6.52±1.93	11.82±11.03	66.70±65.78	2.32±0.38	51.58±7.27
	*a*_*x*_	0.91±0.25	0.19±0.13	0.43±0.43	0.98±0.16	1.36±0.31
Effect on *v*_*m*_	*K*_*v*_	0.38±0.12	0.83±0.06	0.86±0.16	1.14±0.60	0.53±0.08
	*m*_*v*_ (mM or μM)	9.29±5.35	0.33±0.32	1.35 (NS)	1.88±1.73	33.22±17.26
	*a*_*v*_	1.35±1.13	0.36±0.14	0.34±0.20	0.40±0.21	0.66±0.33
Effect on *λ*	*K*_*λ*_	1.76±0.70	NS	NS	1.27±1.13	NS
	*m*_*λ*_ (mM or μM)	2.16±0.29	NS	NS	2.53±1.45	NS
	*a*_*λ*_	2.39±0.99	NS	NS	2.33±2.31	NS
	*EC*_*50,*τ_ (mM or μM)	2.45	0.96	2.20	0.91	21.43
	*τ* (h or d)	0.75	17.31	20.52	0.65	6.54
	*p*-value	<0.001	<0.001	<0.001	<0.001	<0.001
	*B*_*f*_	0.99	1.04	1.00	1.02	1.03
	*A*_*f*_	1.06	1.09	1.18	1.11	1.08
	*R*^*2*^_*adj*_	0.995	0.988	0.984	0.990	0.995

The case of apomyoglobin affected by trehalose is also shown. Statistical values of adjusted coefficient of multiple determination (*R*^*2*^_*adj*_) and *p*-values from Fisher’s F-test (α = 0.05). *B*_*f*_ and *A*_*f*_ are the bias and accuracy factor, respectively. NS: non-significant.

The numerical values of *EC*_*50,τ*_ confirmed the superior antifibrillogenic capacity of the biflavonoid taiwaniaflavone (*EC*_*50,τ*_ = 0.91 μM) compared with the monoflavonoid apigenin (*EC*_*50,τ*_ = 2.45 μM). However, the time τ was quite similar in both cases. This affirmation is consistent with the conclusions reported in the original work conducted by Thapa et al. [16].

### Inhibitory effect of osmolytes on Aβ42 amyloid protein

Different types of molecules have been recently suggested as drug candidates for the treatment of neurodegenerative disorders caused by the anti-aggregation properties of amyloid proteins [49]. One of the most interesting candidates are osmolytes (compounds that protect against the osmotic stress), such as the ectoines that are obtained from aerobic heterotrophic bacteria [50]. The response surface for hydroxyectoine (Figure 4C) shows a behavior that is similar to that obtained with the simulation shown in Figure 2B. Based on the numerical fittings summarized in Table 4, it was statistically demonstrated that osmolytes inhibited the maximum aggregation rate and the maximum fibrillation growth. In contrast, no significant changes in the lag phase were observed. Therefore, both osmolytes effectively blocked the post-nucleation and the elongation stages but not the pre-nuclei formation. In addition, ectoine was much more effective as an antineurotoxic (*EC*_*50,τ*_ = 0.96 mM) than hydroxyectoine (*EC*_*50,τ*_ = 2.20 mM).

### Inhibition of apomyoglobin fibrils formation by trehalose

The effect of trehalose on the apomyoglobin aggregation kinetics was also studied using model (1), and excellent statistical results were obtained from the modeling and the description of the experimental data (Figure 4D and Table 4). As in the previous case, the disaccharide significantly inhibited the two kinetic parameters *v*_*m*_ and *X*_*m*_ without affecting the pre-nucleation phase (*λ*). Nevertheless, its relative efficacy was obtained at a much higher concentration (21.43 mM), and much longer times (6.5 d) were required than compared with the other cases studied.

## Conclusions

In summary, a general bivariate model that combines the logistic equation for the description of kinetics and the Weibull equation for the chemical-concentration response has been proposed for the characterization of the inhibitory effects produced by several chemicals on the growth of the aggregation of amyloid proteins. In all cases, the inhibitory effects on the kinetic parameters were established, and the theoretical response surfaces were in perfect agreement with the selected data. In addition, the recent definition of true and apparent inhibitors reported by Martins [24] can be also evaluated and validated. EGCG at pH 7.4 and a temperature of 37°C was the best option for the reduction of the formation of insulin fibrils (*EC*_*50,τ*_ = 6 μM) but the shortest time (τ) was obtained with methylglyoxal. For the Aβ42 amyloid protein, the biflavonoid taiwaniaflavone produced the highest inhibition response at the lowest dose (0.91 μM) and the shortest time (0.65 h). The bivariate equation was validated using data obtained by two methods based on different chemical phenomena. Although this model can not define the mechanisms of action of chemical inhibitors on protein aggregation, it provides a consistent tool for the comparison of the ability of such compounds in the inhibition of the protein fibrillation process, regardless of the method used for its determination, and is a first step for the optimization of the *in vitro* application of them. Further experiments and corresponding modeling should be done to establish its validity for *in vivo* applications of anti-aggregation chemicals.

## Appendix

### Reparameterization of logistic equation

It is well-known the autocatalytic origin of the logistic equation based on the following differential equation:

(A.1) $$\frac{\mathrm{dX}}{\mathrm{dt}}=k_{\mathrm{app}}X\;\left(1-\frac{X}{X_{m}}\right)$$

which, integrated between $X_{0}\to X$ and 0→t gives the explicit form of aggregation growth as a function of the time:

(A.2) $$X=\frac{X_{m}}{1+\exp\left[\ln\;\left(\frac{X_{m}-X_{0}}{X_{0}}\right)-k_{\mathrm{app}}t\right]}$$

The parameter τ is defined as the time required to obtain the semimaximum fibrillation growth (when *X* = *X*_*m*_/2):

(A.3) $$\frac{X_{m}}{2}=\frac{X_{m}}{1+\left(\frac{X_{m}-X_{0}}{X_{0}}\right)\;\exp\;\left[-k_{\mathrm{app}}\tau \right]}\Rightarrow \;\tau =\frac{1}{k_{\mathrm{app}}}\ln\;\left(\frac{X_{m}-X_{0}}{X_{0}}\right)$$

The inflection point (*t* = *t*_*i*_) can be obtained when the second derivative from (A.1) is equal to zero and the abscissa is isolated [31]:

(A.4) $$t_{i}=\tau =\frac{1}{k_{\mathrm{app}}}\ln\;\left(\frac{X_{m}-X_{0}}{X_{0}}\right)$$

The value of aggregation when *t* = *t*_*i*_ is:

(A.5) $$X\left(t_{i}\right)=\frac{X_{m}}{2}$$

The slope in the inflection point (*v*_*m*_) is defined by the following operation:

(A.6) $$\begin{array}{l} v_{m}={\left(\frac{\mathrm{dX}}{\mathrm{dt}}\right|}_{t=t_{i}}=\frac{X_{m}k_{\mathrm{app}}\exp\;\left[\ln\;\left(\frac{X_{m}-X_{0}}{X_{0}}\right)-k_{\mathrm{app}}t_{i}\right]}{{\left[1+\exp\;\left[\ln\;\left(\frac{X_{m}-X_{0}}{X_{0}}\right)-k_{\mathrm{app}}t_{i}\right]\right]}^{2}}\\ \;=\frac{X_{m}k_{\mathrm{app}}}{4} \end{array}$$

The lag phase (*λ*) or pre-nucleation phase is defined as the intersection of the tangent at the inflection point with the abscissa (Figure 1):

$$R=X\left(t_{i}\right)+v_{m}\left(L-t_{i}\right)$$

with *L* = *λ* when *R* = 0:

(A.7) $$\lambda =t_{i}-\frac{X\left(t_{i}\right)}{v_{m}}=\tau -\frac{X_{m}}{2v_{m}}=\tau -\frac{2}{k_{\mathrm{app}}}$$

Reorganizing terms, two reparameterized functions can be defined:

(A.8) $$X=\frac{X_{m}}{1+\exp\;\left[2+\frac{4v_{m}}{X_{m}}\left(\lambda -t\right)\right]}$$

(A.9) $$X=\frac{X_{m}}{1+\exp\;\left[\frac{4v_{m}}{X_{m}}\left(\tau -t\right)\right]}$$

When parameters from both equations are influenced by chemical agent concentration, they can be rewritten as follows:

(A.10) $$X=\frac{X_{m•}}{1+\exp\;\left[2+\frac{4v_{m•}}{X_{m•}}\left(\lambda_{•}-t\right)\right]}$$

(A.11) $$X=\frac{X_{m•}}{1+\exp\;\left[\frac{4v_{m•}}{X_{m•}}\left(\tau_{•}-t\right)\right]}$$

On the other hand, the calculation of the time τ for *EC*_*50,*τ_ is obtained by the following formula:

(A.12) $$\begin{array}{l} \frac{X_{m•}}{4}=\frac{X_{m•}}{1+\exp\left[\frac{4v_{m•}}{X_{m•}}\left(\tau_{•}-\tau \right)\right]}\Rightarrow \tau \\ \;=\tau_{•}-\frac{X_{m•}\ln3}{4v_{m•}} \end{array}$$

*Weibull equation and bivariate model (*1*)*

The most interesting form of Weibull equation for dose-response modelling is expressed as follows [35]:

(A.13) $$Y=Y_{m}\left\{1-\exp\;\left[-\ln2\;{\left(\frac{C}{m}\right)}^{a}\right]\right\}$$

where, *Y* is the response and *Y*_*m*_ the maximum response, *m* is the concentration corresponding to the semi-maximum response, *C* is the concentration and *a* is a shape parameter related to the maximum slope of the response.

This equation can be modified according with the graphical tendencies of chemicals effects (Figure 1) on the aggregation parameters [38]:

(A.14) $$\theta =\theta_{0}\left(1-Y_{\theta }\right)\;\mathrm{or}\;\theta =\theta_{0}\left(1+Y_{\theta }\right)$$

Thus, decrease of *v*_*m*_ and *X*_*m*_ and increase of *λ* and τ were the characteristic responses observed for the inhibition of amyloid protein fibrillation kinetics induced by chemicals:

(A.15) $$\begin{array}{l} X_{m•}=X_{m}\left\{1-K_{x}\left[1-\exp\left(-\ln2{\left(\frac{C}{m_{x}}\right)}^{a_{x}}\right)\right]\right\}\\ v_{m•}=v_{m}\left\{1-K_{v}\left[1-\exp\left(-\ln2{\left(\frac{C}{m_{v}}\right)}^{a_{v}}\right)\right]\right\}\\ \lambda_{•}=\lambda \left\{1+K_{\lambda }\left[1-\exp\left(-\ln2{\left(\frac{C}{m_{\lambda }}\right)}^{a_{\lambda }}\right)\right]\right\}\\ \tau_{•}=\tau \left\{1+K_{\tau }\left[1-\exp\left(-\ln2{\left(\frac{C}{m_{\tau }}\right)}^{a_{\tau }}\right)\right]\right\} \end{array}$$

When the equations (A.15) are inserting directly on equation (A.10) or (A.11), the bivariate model (1) is obtained.

## Competing interests

The author declares that he has no competing interests.

## Pre-publication history

The pre-publication history for this paper can be accessed here:

http://www.biomedcentral.com/2050-6511/15/9/prepub

## Supplementary Material

Additional file 1 Excel spreadsheet used for modeling the Ab42amyloid apigenin case. Click here for file

## References

[B1] Bucciantini M Giannoni E Chiti F Baroni F Formigli L Zurdo J Taddei N Ramponi G Dobson CM Stefani M Inherent toxicity of aggregates implies a common mechanism for protein misfolding diseases Nature 2002 416 507 511 11932737 10.1038/416507a

[B2] Chiti F Dobson CM Protein misfolding, functional amyloid, and human disease Annu Rev Biochem 2006 75 333 366 16756495 10.1146/annurev.biochem.75.101304.123901

[B3] Stefani M Dobson CM Protein aggregation and aggregate toxicity: new insights into protein folding, misfolding diseases, and biological evolution J Mol Med 2003 81 678 699 12942175 10.1007/s00109-003-0464-5

[B4] Uversky VN Fink AL Conformational constraints for amyloid fibrillation: the importance of being unfolded Biochim Biophys Acta 2004 1698 131 153 15134647 10.1016/j.bbapap.2003.12.008

[B5] Wang SSS Good TA An overview of Alzheimer's disease J Chin Inst Chem Eng 2005 36 533 559

[B6] Cromwell MEM Hilario E Jacobson F Protein aggregation and bioprocessing AAPS J 2006 8 E572 E579 17025275 10.1208/aapsj080366 PMC2761064

[B7] Rosenberg AS Effects of protein aggregates: an immunologic perspective AAPS J 2006 8 E501 E507 17025268 10.1208/aapsj080359 PMC2761057

[B8] Knowles TP Fitzptrick AW Meehan S Mott HR Vendruscolo M Dobson CM Welland ME Role of intermolecular forces in defining material properties of protein nanofibrils Science 2007 318 1900 1903 18096801 10.1126/science.1150057

[B9] Nelson R Eisenberg D Recent atomic models of amyloid fibril structure Curr Opin Struct Biol 2006 16 260 265 16563741 10.1016/j.sbi.2006.03.007

[B10] Ross CA Poirier MA What is the role of protein aggregation in neurodegeneration? Nat Rev Mol Cell Biol 2005 6 891 898 16167052 10.1038/nrm1742

[B11] Mauro M Craparo EF Podesta A Bulone D Carrotta R Martorana V Tiana G San Biagio PL Kinetics of different processes in human insulin amyloid formation J Mol Biol 2007 366 258 274 17157312 10.1016/j.jmb.2006.11.008

[B12] Loksztejn A Dzwolak W Vortex-induced formation of insulin amyloid superstructures probed by time-lapse atomic force microscopy and circular dichroism spectroscopy J Mol Biol 2010 395 643 655 19891974 10.1016/j.jmb.2009.10.065

[B13] Slusky V Tamada JA Klibanov AM Langer R Kinetics of insulin aggregation in aqueous solutions upon agitation in the presence of hydrophobic surfaces Proc Natl Acad Sci U S A 1991 88 9377 9381 1946348 10.1073/pnas.88.21.9377 PMC52720

[B14] Dische FE Wernstedt C Westermark GT Westermark P Pepys MB Rennie JA Gilbey SG Watkins PJ Insulin as an amyloid-fibril protein at sites of repeated insulin injections in a diabetic patient Diabetol 1988 31 158 161 10.1007/BF00276849 3286343

[B15] Kanapathipillaia M Lentzenb G Sierksa M Parka CB Ectoine and hydroxyectoine inhibit aggregation and neurotoxicity of Alzheimer’s β-amyloid FEBS Lett 2005 579 4775 4780 16098972 10.1016/j.febslet.2005.07.057

[B16] Thapa A Woo ER Chi EY Sharoar MG Jin HG Shin SY Park IS Biflavonoids are superior to monoflavonoids in inhibiting amyloid-β toxicity and fibrillogenesis via accumulation of nontoxic oligomer-like structures Biochem 2011 50 2445 2455 21322641 10.1021/bi101731d

[B17] Andrews JM Roberts CJ A Lumry-Eyring nucleated polymerization model of protein aggregation kinetics: 1. Aggregation with pre-equilibrated unfolding J Phys Chem B 2007 111 7897 7913 17571872 10.1021/jp070212j

[B18] Bernacki J Murphy RM Model discrimination and mechanistic interpretation of kinetic data in protein aggregation studies Biophys J 2009 96 2871 2887 19348769 10.1016/j.bpj.2008.12.3903 PMC2711288

[B19] Ghosh P Kumar A Datta B Rangacharim V Dynamics of protofibril elongation and association involved in Aβ42 peptide aggregation in Alzheimer’s disease BMC Bioinformatics 2010 11 Suppl 6 S24 20946608 10.1186/1471-2105-11-S6-S24 PMC3724481

[B20] Lee CC Nayak A Sethuraman A Belfort G McRae GJ A three-stage kinetic model of amyloid fibrillation Biophys J 2007 92 3448 3458 17325005 10.1529/biophysj.106.098608 PMC1853138

[B21] Roberts CJ Kinetics of irreversible protein aggregation: Analysis of extended Lumry-Eyring models and implications for predicting protein shelf life J Phys Chem B 2003 107 1194 1207

[B22] Ruzafa D Conejero-Lara F Morel B Modulation of the stability of amyloidogenic precursors by anion binding strongly influences the rate of amyloid nucleation Phys Chem Chem Phys 2013 15 15508 15517 23942905 10.1039/c3cp52313f

[B23] Ruzafa D Morel B Varela L Azuaga AI Conejero-Lara F Characterization of oligomers of heterogeneous size as precursors of amyloid fibril nucleation of an SH3 domain: an experimental kinetics study Plos One 2012 7 e49690 23209591 10.1371/journal.pone.0049690 PMC3507826

[B24] Morris AM Watzky MA Finke RG Protein aggregation kinetics, mechanism, and curve-fitting: a review of the literature Biochim Biophys Acta 2009 1794 375 397 19071235 10.1016/j.bbapap.2008.10.016

[B25] Crespo R Rocha FA Damas AM Martins PM A generic crystallization-like model that describes the kinetics of amyloid fibril formation J Biol Chem 2012 287 30585 30594 22767606 10.1074/jbc.M112.375345 PMC3436372

[B26] Martins PM True and apparent inhibition of amyloid fibril formation Prion 2013 7 136 139 23232498 10.4161/pri.23111 PMC3609120

[B27] Morris AM Watzky MA Agar JN Finke RG Fitting neurological protein aggregation kinetic data via a 2-step, minimal/“Ockham’s Razor” model: the Finke-Watzky mechanism of nucleation followed by autocatalytic surface growth Biochem 2008 47 2413 2427 18247636 10.1021/bi701899y

[B28] Morris AM Finke RG α-Synuclein aggregation variable temperature and variable pH kinetic data: a re-analysis using the Finke–Watzky 2-step model of nucleation and autocatalytic growth Biophys Chem 2009 140 9 15 19101068 10.1016/j.bpc.2008.11.003

[B29] Naiki H Hasegawa K Yamaguchi I Nakamura H Gejyo F Nakakuki K Apolipoprotein E and antioxidants have different mechanisms of inhibiting Alzheimer’s β-Amyloid fibril formation in vitro Biochem 1998 37 17882 17889 9922155 10.1021/bi980550y

[B30] Nielsen L Khurana R Coats A Frokjaer S Brange J Vyas S Uversky VN Fink AL Effect of environmental factors on the kinetics of insulin fibril formation: elucidation of the molecular mechanism Biochem 2001 40 6036 6046 11352739 10.1021/bi002555c

[B31] Wang SH Dong XY Sun Y Effect of (−)-epigallocatechin-3-gallate on human insulin fibrillation/aggregation kinetics Biochem Eng J 2012 63 38 49

[B32] Oliveira LMA Lages A Gomes RA Neves H Família C Coelho AV Quintas A Insulin glycation by methylglyoxal results in native-like aggregation and inhibition of fibril formation BMC Biochem 2011 12 41 21819598 10.1186/1471-2091-12-41 PMC3175161

[B33] Wang SSS Liu KN Han TC Amyloid fibrillation and cytotoxicity of insulin are inhibited by the amphiphilic surfactants Biochim Biophys Acta 2010 1802 519 530 20176106 10.1016/j.bbadis.2010.02.008

[B34] Vilasi S Iannuzzi C Portaccio M Irace G Sirangelo I Effect of trehalose on W7FW14F apomyoglobin and insulin fibrillization: new insight into inhibition activity Biochem 2008 47 1789 1796 18205397 10.1021/bi701530w

[B35] Murado MA Vázquez JA Rial D Beiras R Dose-response modeling with two agents: application to the bioassay of oil and shoreline cleaning agents J Hazardous Materials 2011 185 807 817 10.1016/j.jhazmat.2010.09.092 20970248

[B36] Riobó P Paz B Franco JM Vázquez JA Murado MA Cacho E Mouse bioassay for palytoxin. Specific symptoms and dose-response against dose-death time relationships Food Chem Toxicol 2008 46 2639 2647 18534735 10.1016/j.fct.2008.04.020

[B37] Vázquez JA Murado MA Mathematical tools for objective comparison of microbial cultures Application to evaluation of 15 peptones for lactic acid bacteria productions Biochem Eng J 2008 39 276 287

[B38] Rial D Vázquez JA Murado MA Effects of three heavy metals on the bacteria growth kinetics: a bivariate model for toxicological assessment Appl Microbiol Biotechnol 2011 90 1095 1109 21360150 10.1007/s00253-011-3138-1

[B39] Vázquez JA Durán A Rodríguez-Amado I Prieto MA Rial D Murado MA Evaluation of toxic effects of several carboxylic acids on bacterial growth by toxicodynamic modelling Microb Cell Fact 2011 10 100 22118421 10.1186/1475-2859-10-100 PMC3235065

[B40] Ross T Indices for performance evaluation of predictive models in food microbiology J Appl Bacteriol 1996 81 501 508 8939028 10.1111/j.1365-2672.1996.tb03539.x

[B41] Vázquez JA Murado MA Unstructured mathematical model for biomass, lactic acid and bacteriocin production by lactic acid bacteria in batch fermentation J Chem Technol Biotechnol 2008 83 91 96

[B42] Sabaté R Villar-Piqué A Espargaró A Ventura S Temperature dependence of the aggregation kinetics of Sup35 and Ure2p yeast prions Biomacromolecules 2012 13 474 483 22176525 10.1021/bm201527m

[B43] Fändrich M Absolute correlation between lag time and growth rate in the spontaneous formation of several amyloid-like aggregates and fibrils J Mol Biol 2007 365 1266 1270 17141269 10.1016/j.jmb.2006.11.009

[B44] Vázquez JA Lorenzo JM Fuciños P Franco D Evaluation of non-linear equations to model different animal growths with mono and bisigmoid profiles J Theor Biol 2012 314 95 105 22960569 10.1016/j.jtbi.2012.08.027

[B45] Murado MA González MP Vázquez JA Dose-reponse relationships. An overview a generative model and its application to the verification of descriptive models Enz Microb Technol 2002 31 439 455

[B46] Riobó P Paz B Franco JM Vázquez JA Murado MA Proposal for a simple and sensitive haemolytic assay for palytoxin. Toxicological dynamics, kinetics, ouabain inhibition and thermal stability Harmful Algae 2008 7 415 429

[B47] Shimanouchi T Onishi R Kitaura N Umakoshi H Kuboi R Copper-mediated growth of amyloid β fibrils in the presence of oxidized and negatively charged liposomes J Biosc Bioeng 2011 112 611 615 10.1016/j.jbiosc.2011.08.015 21917513

[B48] Wang SSS Chou SW Liu KN Wu CH Effects of glutathione on amyloid fibrillation of hen egg-white lysozyme Int J Biol Macromol 2009 45 321 329 19699759 10.1016/j.ijbiomac.2009.08.003

[B49] Arora A Ha C Park CB Inhibition of insulin amyloid formation by small stress molecules FEBS Lett 2004 564 121 125 15094052 10.1016/S0014-5793(04)00326-6

[B50] Vorob’eva LI Stressors, stress reactions, and survival of bacteria: a review Appl Biochem Microbiol 2004 40 261 269 15283326

